# Environmental Chemical Exposomics and Metabolomics in Toxicology: The Latest Updates

**DOI:** 10.3390/toxics12090647

**Published:** 2024-09-04

**Authors:** Minjian Chen

**Affiliations:** 1 State Key Laboratory of Reproductive Medicine and Offspring Health, Center for Global Health, School of Public Health, Nanjing Medical University, Nanjing 211166, China; minjianchen@njmu.edu.cn; 2 Key Laboratory of Modern Toxicology of Ministry of Education, School of Public Health, Nanjing Medical University, Nanjing 211166, China

## 1. Introduction

This Editorial introduces the Special Issue titled “State-of-the-Art Environmental Chemical Exposomics and Metabolomics”. Due to the complexity and variability of human exposures, there is increasing interest in exploring the relationship between environmental exposures and human health from a more comprehensive perspective. This interest has spurred the development of the concept of chemical exposomes, which aims to thoroughly evaluate chemical exposures and their associated risks [1]. Metabolomics, which focuses on the high-throughput detection of endogenous chemicals, systematically identifies small molecule substrates, intermediates, and products of cellular metabolism, which are considered to be closest to the phenotype and provide important information for understanding physiological and pathological processes, making it highly significant in toxicology [2]. By integrating environmental chemical exposomics with metabolomics, researchers can gain valuable information for identifying key chemicals that lead to adverse outcomes and their toxic metabolic signatures, thereby clarifying the potentially harmful effects of these chemical exposures and the underlying mechanisms involved.

This Special Issue contains 13 articles—12 research papers and 1 review. The research papers focus on developing new methods for detecting specific chemical exposomes, exploring new aspects of exposome-related toxicity, applying metabolomics, and integrating metabolic information with exposomics and other omics across different biological layers in toxicology. The review article focuses on a neurotoxicity-related system linked to metabolism targeted by multiple chemicals. These articles advance our understanding of environmental chemical exposomes and metabolomics within toxicology.

## 2. An Overview of Published Articles

Detecting chemicals related to the exposome involves the development of new methods, which is both necessary and challenging. Mycotoxins, secondary metabolites produced by molds and fungi, are a major concern due to their potential contamination of agricultural products and various foods. These toxins pose risks to human health and livestock and are an integral part of the exposome. For external exposure, Ning et al. (contribution 1) introduced a novel, convenient, and sensitive quantitative method for detecting trace levels of seven type B trichothecene mycotoxins in preprocessed whole-grain foods. This method was applied to 160 food samples from China, revealing mycotoxin contamination in 70% of the samples, with whole-wheat dumpling wrappers exhibiting the highest contamination rate, posing a significant health threat. For internal exposure, Ning et al. (contribution 2) developed a highly sensitive and comprehensive analytical method for quantifying 67 mycotoxins in human plasma using mass spectrometry. This method identified 40 mycotoxins, including 24 emerging ones, in 184 plasma samples from both infertile and healthy males. Notably, infertile males had significantly higher levels of multiple mycotoxins, especially ochratoxins A and B and citrinin; this highlights the need for further research investigating the link between mycotoxins and male infertility.

This Special Issue presents four articles addressing novel exposures within the chemical exposome—two focus on nanomaterials, while the other two concentrate on micro/nanoplastics. These studies employed metabolomics and integrated information from other omics and biological layers, such as transcriptomics, to provide new insights into the pulmonary and reproductive toxicities associated with these materials. Liu et al. (contribution 3) examined the effects of polyvinyl chloride microplastics (PVC-MPs) on human lung cells, finding that PVC-MPs reduced BEAS-2B cell viability. Bioinformatics analysis, through analyzing the changes in the metabolome and transcriptome, showed that PVC-MPs disrupted lipid metabolic pathways including glycerophospholipid metabolism, glycerolipid metabolism, and sphingolipid metabolism, affecting 530 genes and 3768 metabolites. Yao et al. (contribution 4) reported the cytotoxicity of tungsten ions in BEAS-2B cells, including chromatin condensation and organelle damage; however, they noted that the ion chelator EDTA-2Na reduced these effects, suggesting potential therapeutic strategies. Lv et al. (contribution 5) investigated the embryotoxic effects of polystyrene nanoplastics (PS-NPs) in mice and human trophoblastic cells, finding that 30 nm PS-NPs caused abnormal embryonic development, a reduced placental weight, and altered the p38 MAPK pathway. PS-NPs also impaired HTR-8/SVneo cell vitality and migration. Li et al. (contribution 6) assessed the impact of titanium dioxide nanoparticles (TiO_2_ NPs) on pregnant rats and their offspring. The authors found maternal preeclampsia-like symptoms, fetal growth restriction, and impaired trophoblastic cell invasion into the endometrium due to autophagy. Another significant component of the chemical exposome featured in this Special Issue is cigarette smoke. Exosomes are natural carriers secreted by cells to transport a diverse range of cargo, including numerous biological molecules such as proteins, nucleic acids, and metabolites, between different cells. Liang et al. (contribution 7) explored the effects of cigarette smoke, revealing that smoke extract enhanced stemness and epithelial-to-mesenchymal transition (EMT) in gastric cancer cells, finding that exosomal circ0000670 promoted gastric cancer development via the Wnt/β-catenin pathway.

Metabolomics and changes in endogenous metabolites provide insights that are close to pathological states. Therefore, focusing on metabolite information, their regulation, and effects is an effective way to study disease biomarkers, pathogenesis, and chemical toxicities. Xu et al. (contribution 8) reported the metabolomic characteristics of papillary thyroid cancer (PTC) by analyzing serum and tissue between patients with and without lymph node metastasis using UPLC-Q-Exactive mass spectrometry, offering new perspectives on diagnosing and treating PTC. This Special Issue includes studies on the toxicity mechanisms related to endogenous metabolites, integrating metabolome data with exposome, transcriptome, and microbiome information, focusing primarily on amino acid, lipid, and nucleotide acid metabolism. Zhou et al. (contribution 9) examined the metabolic toxicity of molybdate at human-relevant exposure levels using an integration of inductively coupled plasma–mass spectrometry (ICP-MS)-based elementomics (element part of exposomics) and UPLC-Q-Exactive mass spectrometry-based metabolomics. The research reported that molybdate disrupted amino acid and lipid metabolism, potentially through altered cadmium levels, supported by human evidence. Zhang et al. (contribution 10) investigated diquat (DQ)-induced kidney damage using multi-omics analysis including transcriptomic, proteomic, and metabolomic analyses in a mouse model. They identified 869 genes, 351 proteins, and 96 metabolites affected by DQ treatment and found that DQ-induced kidney damage involved the dysregulation of the PPAR pathway and elevated Hmgcs2 expression and 3-hydroxybutyric acid levels. Liu et al. (contribution 11) investigated the neurodevelopmental toxicity of bisphenol A (BPA) in zebrafish larvae during their early development. BPA exposure from the cleavage to the segmentation stages significantly impaired the larvae’s spontaneous movement. Transcriptomic analysis revealed 131 differentially expressed genes, and further examination showed that guanine deaminase mRNA levels and enzyme activity were notably decreased in BPA-exposed larvae. Guanine deaminase, which catalyzes the conversion of metabolite guanine to xanthine, is essential for degrading guanine-containing nucleotides. The researchers restored guanine deaminase levels through microinjection, which improved locomotor activity, suggesting that guanine deaminase may be a key target for BPA toxicity. Wu et al. (contribution 12) investigated metabolome and gut microbiome changes associated with acute myeloid leukemia (AML) in humans and mice, finding that the Carnosine–Histidine metabolic pathway may be crucial in AML progression, with gut microbiota like *Peptococcaceae* and *Campylobacteraceae* potentially being involved. The antioxidant system components are integral to metabolic processes; thus, studying how chemicals impact these systems connects chemical exposure with metabolism and disease. In this Special Issue, Xu et al. (contribution 13) provide a comprehensive overview of current toxicology research. They report that exposure to various chemicals, including morphine, metals, and methylglyoxal, disrupts the homeostasis of the thioredoxin system—which comprises NADPH, oxidoreductase thioredoxin, and thioredoxin reductase—leading to neurological injury. Conversely, resveratrol and lysergic acid sulfide have been found to alleviate chemical-induced nerve damage. The authors also discuss the underlying mechanisms, offering a foundational reference for future studies on the complex relationship between the thioredoxin system and chemical-induced nerve injury.

## 3. Conclusions

Continuously improving chemical exposome evaluation methods, including developing detection methods for individual internal and external exposures to emerging pollutants, continuously improving metabolomics research in detection methods, research technologies and integration with other omics, and continuously conducting database construction and data mining of exposome and metabolome based on big data and artificial intelligence are research directions of environmental chemical exposomics and metabolomics that need to be carried out in depth. The articles in this Special Issue offer new perspectives on environmental chemical exposome and metabolome research in toxicology, including advancements in detection method development, toxicity, and metabolic mechanism exploration, which enhances our understanding in this field and serves as a foundation for future research and policy making.
